# Diffusion tensor imaging tractography in the one-humped camel (*Camelus dromedarius*) brain

**DOI:** 10.3389/fvets.2023.1231421

**Published:** 2023-08-15

**Authors:** Benjamin Cartiaux, Abdelkader Amara, Ninon Pailloux, Romain Paumier, Atef Malek, Kefya Elmehatli, Souhir Kachout, Boubaker Bensmida, Charles Montel, Germain Arribarat, Giovanni Mogicato

**Affiliations:** ^1^Toulouse Neuroimaging Center, University of Toulouse Paul Sabatier-INSERM-ENVT, Toulouse, France; ^2^Department of Pathology, University of La Manouba, Sidi Thabet, Tunisia; ^3^Department of Nutrition, University of La Manouba, Sidi Thabet, Tunisia; ^4^Regional Commissariat for Agricultural Development, Tataouine, Tunisia; ^5^Toulouse Neuroimaging Center, University of Toulouse Paul Sabatier-INSERM, Toulouse, France

**Keywords:** MRI, tractography, one-humped camel, neuroanatomy, brain

## Abstract

**Introduction:**

Tractography is a technique used to trace the pathways of the brain using noninvasive diffusion tensor imaging (DTI) data. It is becoming increasingly popular for investigating the brains of domestic mammals and other animals with myelinated fibers but the principle of DTI can also apply for those with unmyelinated fibers. In the case of camels, DTI tractography is a promising method for enhancing current knowledge of the brain’s structural connectivity and identifying white-matter tract changes potentially linked to neurodegenerative pathologies. The present study was therefore designed to describe representative white-matter tracts in the one-humped camel DTI tractography.

**Methods:**

*Post mortem* DTI was used to obtain images of two one-humped camel brains using a 3 Tesla system. T2-weighted images were also acquired to identify regions of interest for each fiber tract and a fiber dissection technique was used to complement the DT images. The main association, commissural, and projection fibers were reconstructed and superimposed on T2-weighted images or fractional anisotropy maps.

**Results:**

The results of the present study show the reconstruction of the most representative tracts, ie the cingulum, the corpus callosum and the internal capsule, in the one-humped camel brain using DTI data acquired post mortem. These DTI results were compared to those from fiber dissection.

**Discussion:**

Anatomy of the cingulum, corpus callosum and internal capsule correlates well with the description in anatomical textbooks and appears to be similar to fibers describe in large animals. Further research will be required to improve and validate these findings and to generate a tractography atlas based on MRI and histological data, as such an atlas would be a valuable resource for future neuroimaging research.

## Introduction

Diffusion tensor imaging (DTI) is a popular MRI technique that is extensively used in brain research to describe the orientation of white-matter fibers (1). It was first introduced in 1994 (2). DTI measures the impact of tissue architecture on the diffusion-weighted signal by examining the motion of water molecules. The procedure of fiber tracking, known as tractography, allows for the virtual dissection and three-dimensional representation of white-matter tracts (3).

The distribution of action potentials is modulated by white matter, which functions as a relay system and coordinates communication among various brain regions. White matter tracts in the brain can be categorized into three types: association, commissural, and projection fibers. Association fibers connect different regions within the same cerebral hemisphere and can be classified as short association fibers, which link adjacent gyri, and long association fibers, which establish connections between more distant areas. One of the important functions of association tracts is linking perceptual and memory centers in the brain. Commissural fibers are axons that connect the two hemispheres of the brain. These fibers form tracts such as the corpus callosum, the anterior commissure, and the posterior commissure. Commissural tracts facilitate communication between the left and right sides of the cerebrum and play a role in both homologous and heterotopic associations. Projection fibers encompass both efferent and afferent fibers that connect the cortex with the lower regions of the brain and the spinal cord. They serve as conduits for information transmission between the cerebrum and the rest of the body (4).

DTI tractography is extensively used in the field of brain research, as a means of measuring white-matter integrity. It has considerable potential in terms of both diagnosis and prognosis for a number of brain pathologies, including brain tumors, neurodegenerative diseases, and stroke (5–15). With the growing availability of high field-strength MRI (1.5 and 3 tesla) in veterinary facilities (16–19), this technique is being increasingly used to study white-matter anatomy (16, 20–22) and structural connectivity (23–26) in domestic mammals such as dogs (16, 21, 23) cats (17, 24–26), ferrets (24), sheep (20, 27–29) and horses (19, 22) but also in other animal species such as reptiles (30), birds, cetaceans (31) and rodents (32).

MRI scanners designed for human medicine can also be used to examine large animal brains. Large animals can spontaneously develop a range of typically human brain diseases, not least Alzheimer’s disease (33, 34), along with Parkinson’s disease (35, 36), lysosomal storage diseases (37, 38), and gliomas (39), so these imaging data could be extremely useful both in experimental research and in veterinary medicine. Especially, in experimental research, the use of large animal models, in addition to murine models might be useful to use innovative therapies in human due to their large and convoluted brain, closer to human than murine and the spontaneous development of the disease.

Nevertheless, DTI tractography cannot be validated in large animals without a thorough knowledge of their white-matter tract morphology. A recent study provided detailed anatomical descriptions of the main association, commissural, and projection fibers in dogs, cats, and horses using a fiber dissection technique (40). However, although MRI of the one-humped camel [*Camelus dromedarius* (41)] brain under a 1 or 1.5 T field has been shown to be possible (42–46), the spatial anatomy of white-matter tracts in this species, which can develop poorly understood neurodegenerative diseases such as prion disease (47), Dubduba syndrome (48), and poliencephalomacia (49, 50), has not yet been complemented by DTI tractography. Researchers could use the latter to pinpoint damage in specific white-matter tracts. This damage could then be correlated with clinical symptoms (47–50). Furthermore, the use of DTI tractography in healthy individuals could enhance current knowledge about the camel brain’s anatomy and functioning, based on mapping of its structural connectivity.

The present study was intended to use DTI tractography to characterize the one-humped camel brain fiber bundles. In this context the aim of this study was to investigate the reconstruction of the most representative tracts that connect one part of the brain to another within the white matter, namely the cingulum (belonging to association fibers), corpus callosum (belonging to commissural fibers), and internal capsule (belonging to projection fibers). To this end, a fiber dissection technique and T2-weighted images were used in conjunction with 3 T MRI and the fiber tracking results were compared to the description of these tracts in the literature.

## Materials and methods

### Animal sampling and ethical statement

Two cadaver heads of healthy adult one-humped camels were used in this study. These heads were collected directly from a slaughterhouse (Tataouine, Tunisia). Immediately after slaughter, the brains were extracted from the skull and fixed for 1 month in 10% formalin solution. All experimental procedures were performed in accordance with the relevant guidelines and regulations, and approved by an institutional review board (Comité d’Ethique Science et Santé Animales - Toulouse – France).

### MRI acquisition and preprocessing

MRI scans were performed at the Toulouse Institute for Brain Sciences, using a high-field 3.0 T magnet Philips ACHIEVA dStream, with two dStream flex M coils for signal reception. Twenty-four hours prior to MRI acquisition, the brain was rinsed with water and submerged in a saline solution. Immediately beforehand, the brain was placed in a zip-locked hermetic plastic bag (i.e., MRI-compatible container) that was then filled with saline solution. The bag was gently shaken to dislodge any air bubbles, after which it was sealed and lowered into a foam mold. Balls of cottonwool were used to fill any spaces between the bag and the mold, to ensure that it did not move during acquisition (51). Moreover the brain was positioned laying on the ventral aspect in the MRI.

The primary challenge in post-mortem imaging lies in the effect of tissue fixation, commonly using formalin, which can change tissue properties, including alterations in relaxation times like T1 and T2. These changes make interpreting and quantitatively analyzing T1-w images difficult, as the signal intensities may not accurately represent tissue characteristics or allow for reliable comparisons with *in vivo* imaging data. Therefore, in this post-mortem imaging study, we opted for other sequences like T2-weighted imaging (T2-w) or diffusion-weighted imaging (DWI). These sequences are less impacted by the changes caused by formalin fixation, offering more reliable and interpretable information.

The imaging protocol comprised T2-weighted images using a spin-echo sequence (repetition time: 286 ms; echo time: 1500 ms; voxel size: 1 × 1 × 1 mm; matrix: 240 × 240 × 180) and diffusion-weighted images using a spin-echo sequence (repetition time: 11.5 s; echo time: 76.1 ms; flip angle: 90°; voxel size: 1.97 × 1.97 × 2 mm; matrix: 112 × 112 × 48; 64 independent directions; *b*-value: 3000 s/mm^2^). The acquisition time was 12 min and was repeated five times for averaging, for a total acquisition time of 1 h. We applied an LPCA filter combined with a Rician noise model (52) on MATLAB (MathWorks, Natick MA, United States) to denoise the raw diffusion-weighted data. Using DSI studio (53) we then corrected these data for geometric distortion caused by eddy currents. We implemented the C++ ANTs toolkit (54) to register the T2-weighted images to b0 images for the purpose of anatomical referencing and three-dimensional rendering of the brain.

### DTI reconstruction

For the purpose of the present study, DTI reconstruction was performed on DSI studio, following Basser’s method (55). To model the diffusion, we used the diffusion tensor model described by Basser (56), which is derived from a three-dimensional model of Gaussian diffusion displacement.


$$D=\left(\begin{array}{ccc} D_{xx} & D_{xy} & D_{xz}\\ D_{yx} & D_{yy} & D_{yz}\\ D_{zx} & D_{zy} & D_{zz} \end{array}\right)$$

D is calculated for each separate voxel, using the b0 reference image and diffusion-weighted images. The matrix (D) is then diagonalized, yielding the three eigenvalues $\lambda_{1}\lambda_{2}\;\lambda_{3}$ and three eigenvectors $v_{1}v_{2}\;v_{3}$ required for the diffusion tensor to be visualized and described as an ellipsoid.

### Mean diffusivity, fractional anisotropy, and red, green and blue channels

The three eigenvalues are averaged to obtain MD. This generates a parametric diffusivity map for each voxel, but without the direction of diffusion. Calculating MD allows fractional anisotropy (FA) to be retrieved. FA ranges between 0 (voxel where diffusion is totally isotropic) and 1 (anisotropic voxel where one direction is preponderant).


$$FA=\sqrt{\frac{3}{2}}\;\sqrt{\frac{{\left(\lambda_{1}-MD\right)}^{2}+{\left(\lambda_{2}-MD\right)}^{2}+{\left(\lambda_{3}-MD\right)}^{2}}{\lambda_{1}{\;}^{2}+\lambda_{2}{\;}^{2}+\lambda_{3}{\;}^{2}}}$$

Water diffusion is more extensive along the white-matter tracts because it is easier than passing through them that’s why voxels containing fibers have high FA values. We assigned the three values of the first eigenvector $\left(v_{1}\right)$ to the red, green and blue channels. This produced an image where each color represented a specific fiber orientation: right–left (red), ventral-dorsal (green), and rostral-caudal (blue).

### Tractography

To achieve the most valid connection, we opted for deterministic tracking (53), rather than probabilistic tracking. We have chosen a deterministic tractography approach based on two main reasons: its simplicity, speed, and the clear fiber orientation it provides. Firstly, deterministic tractography, by following the principal diffusion direction in each voxel, offers a straightforward, computationally efficient and fast approach. This is particularly valuable in situations requiring prompt analysis or when computational resources are limited. Secondly, this method enables clear representation of fiber orientations by directly estimating fiber pathways, proving useful in visualizing major neural tracts. However, while acknowledging the benefits of probabilistic tractography, our current acquisition does not meet the requirements for this method, hence our choice for the deterministic approach.

### Parameter summary

For post-mortem deterministic tractography, we adjust three main parameters: the fractional anisotropy (FA) threshold, the angle degree, and the step size. The FA threshold is typically lowered to account for potential post-mortem changes in tissue properties. The angle degree is often increased to allow more flexible curvature of fiber pathways, considering possible alterations in fiber orientation post-mortem. Lastly, a larger step size is preferred to mitigate the impact of noise and inaccuracies in the data while still capturing the general tract trajectory. The parameters used for post-mortem DTI are: FA threshold = 0.15, angle degree = 40 degrees and step size = 1.5 mm.

### Delineation of regions of interest and regions of avoidance

Delineation of regions of interest (ROIs) and regions of avoidance was performed manually for each tract. We targeted both median and transversal planes for the different fibers and delineated additional ROIs to segregate fibers of interest. In determining the placement of ROIs and regions of avoidance, we used several points of reference. These included T2-weighted images, FA and tensor maps, which provided valuable data regarding fiber density, tissue contrast, and the anisotropic properties of the tissue. We also referred to anatomical descriptions of T2-weighted images of the human brain, as detailed in reference (57). These images were particularly useful in identifying key anatomical landmarks that guided ROI delineation. Equine and human tractography atlases (3) further supplemented our process by offering established maps of fiber tracts, thereby serving as a key reference for tracing and distinguishing between different fiber pathways. In certain cases, ROIs were used as seeds to improve tractography results. This strategy allowed for a more focused analysis by tracking fibers specifically emanating from or converging into our ROIs, thereby improving the accuracy and reliability of our results.

### Fiber dissection technique

After MRI acquisition, we used Klinger’s modified method for brain fixation and fiber dissection (58, 59). The dissection techniques followed the already reported ones (40, 60). This method involves the freezing and thawing of brain tissue, where white-matter fibers are gradually peeled away to expose the white-matter tracts.

## Results

### T2-weighted images

**Figure 1:**
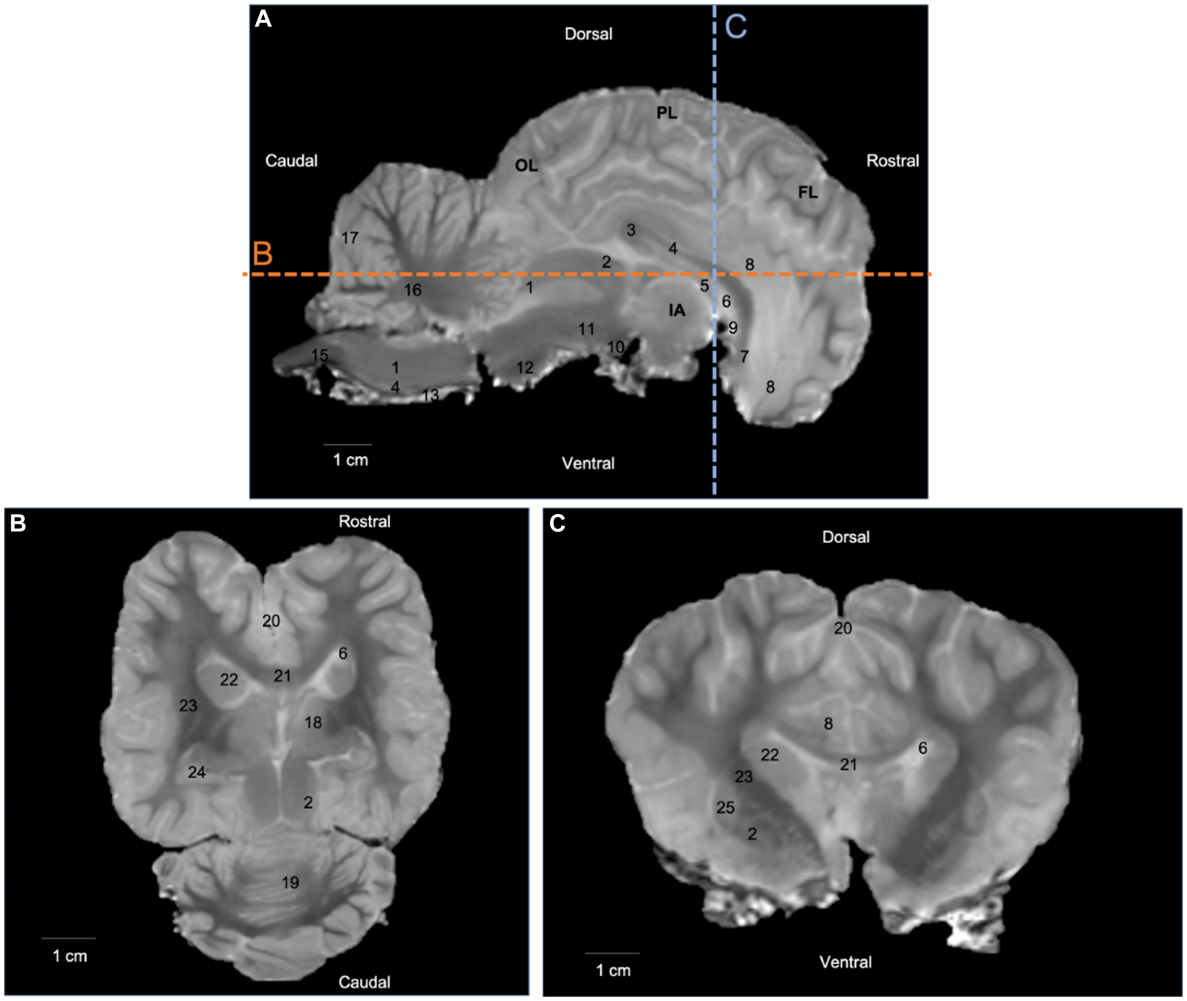
T2-weighted images in **(A)** median plane, **(B)** dorsal plane, and **(C)** transversal plane. Lines b and c on the median plane correspond to the section planes. OL: occipital lobe; PL: parietal lobe; FL: frontal lobe; IA: interthalamic adhesion; 1: caudal colliculus; 2: rostral colliculus; 3: corpus callosum (splenium); 4: corpus callosum (body); 5: fornix; 6: lateral ventricle; 7: corpus callosum (genu); 8: cingulate gyrus; 9: rostral commissure; 10: mammillary body; 11: cerebral peduncle; 12: pons; 13: pyramidal tract; 14: medulla oblongata; 15: spinal cord; 16: cerebellar medulla; 17: cerebellar cortex; 18: thalamus; 19: cerebellum (vermis); 20: longitudinal fissure; 21: corpus callosum; 22: caudate nucleus; 23: internal capsule; 24: hippocampus; 25: putamen; 26: globus pallidus.

T2-weighted images provided good discrimination between white and gray matter, and were useful for anatomical reference, three-dimensional rendering of the brain, and ROI placement. On T2-weighted images, the white matter was hypointense to grey matter. Relevant anatomical structures were identified and labelled in three planes (median, dorsal and transversal). Additional sections in dorsal and transversal planes are available in a Supplementary material. The T2-weighted images allow for the identification of white matter structures such as the corpus callosum on Figures 1B,C (number 21) and its different parts on Figure 1A (numbers 3, 4 and 7), the fornix on Figure 1A (number 5), the rostral commissure on Figure 1A (number 9), the internal capsule on Figures 1B,C (number 23) and the cerbellar medulla on Figure 1A (number 16). Similarly, some gray matter structures of the brain were easily identified on T2-weighted images (Figure 1).

### DTI tractography

We were able to successfully reconstruct three fiber subgroups of groups typically examined in tractography studies, namely association, commissural, and projection: the cingulum, the corpus callosum and the internal capsule.

### Association fibers

**Figure 2:**
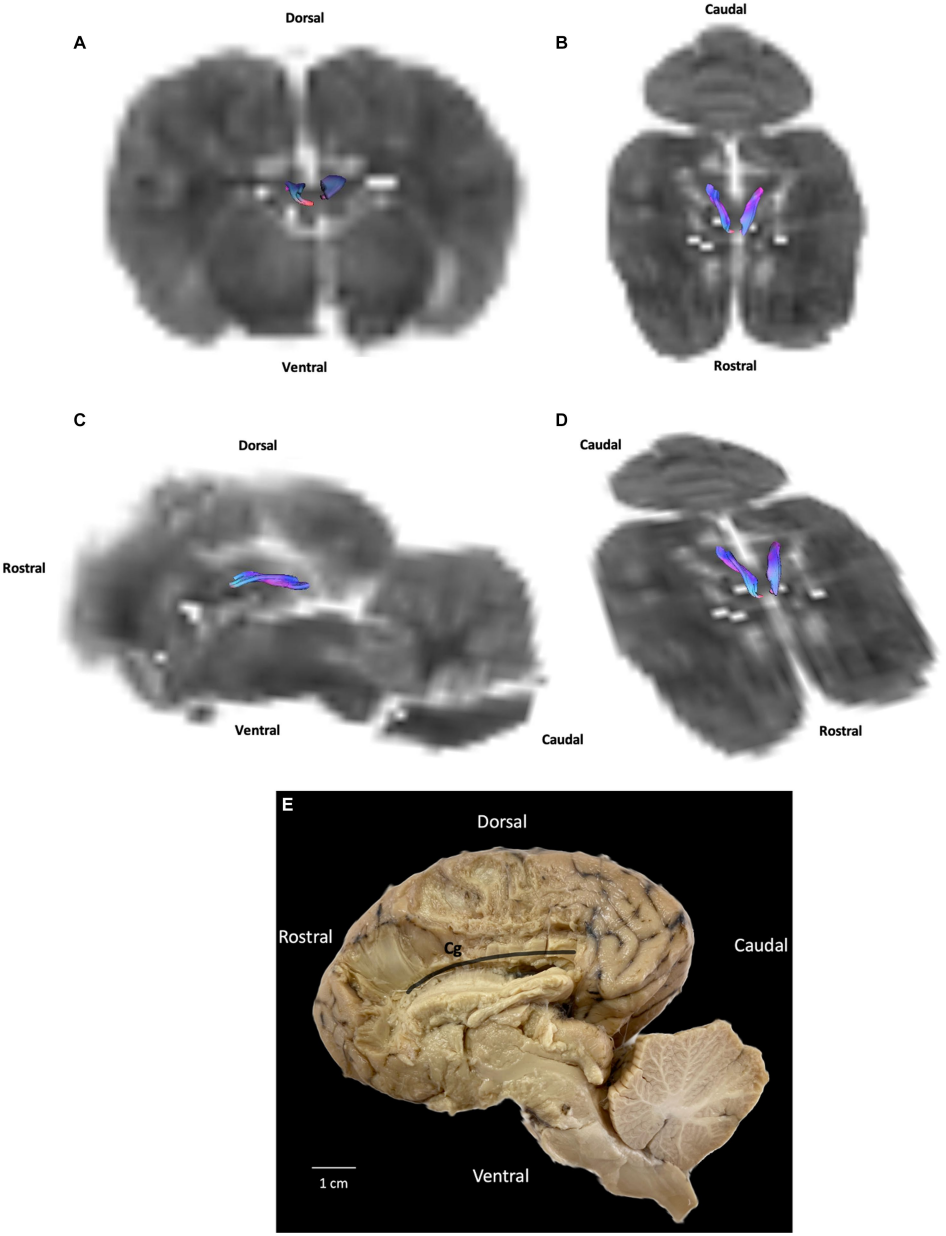
Example of association fibers (cingulum): **(A)** transversal plane – rostral view; **(B)** dorsal plane – dorsal view; **(C)** sagittal plane – left view; **(D)** oblique plane – dorsal view and **(E)** fiber dissection (underlined in black) using Klingler’s method. Cg: cingulum. All tracts are overlaid on the b0 image obtained by DTI.

**Figure 3:**
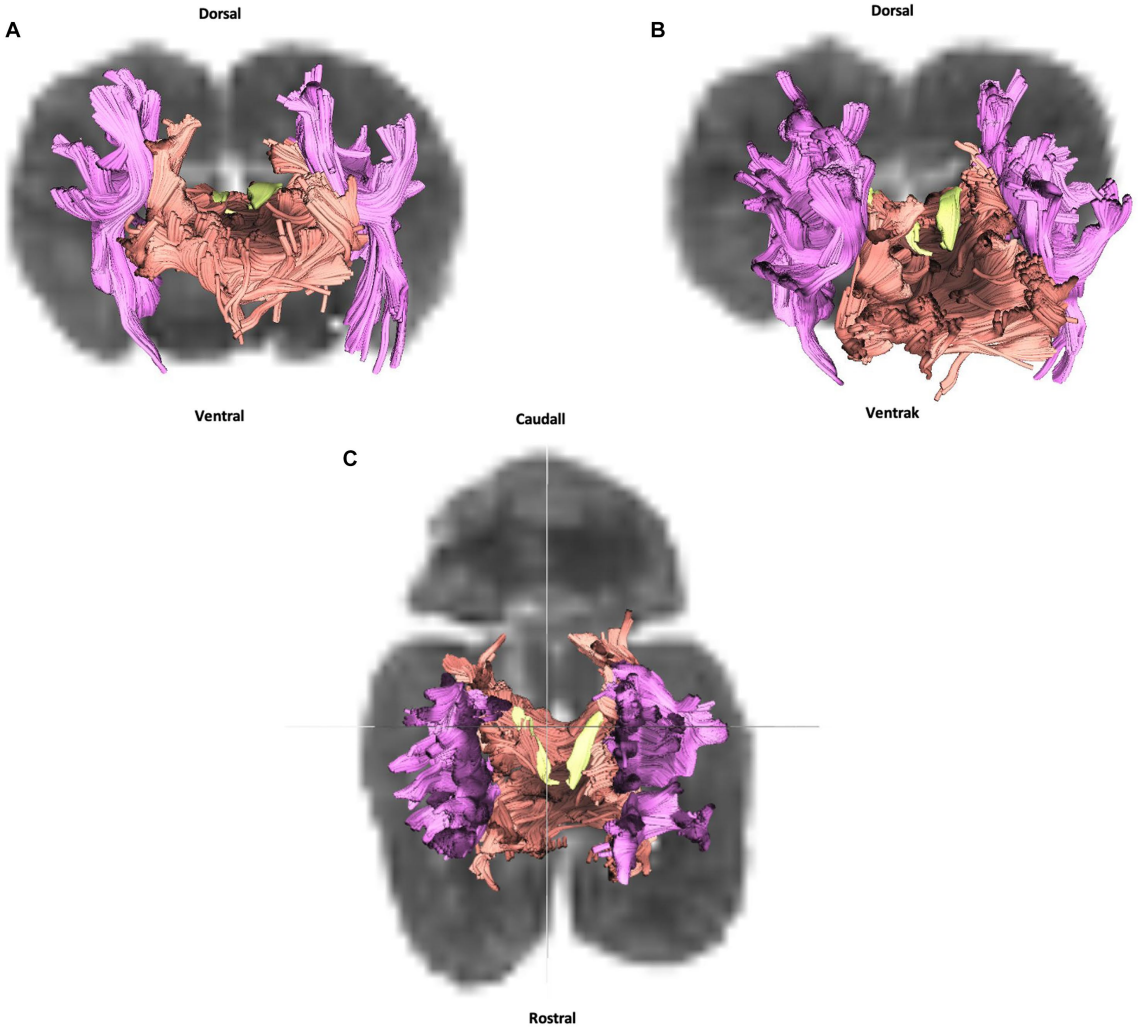
Cingulum (in yellow), corpus callosum (in orange) and internal capsule (in purple): **(A)** transversal plane – rostral view; **(B)** oblique plane – rostral view and **(C)** dorsal plane – dorsal view. All tracts are overlaid on the b0 image obtained by DTI.

Association fibers link gyri (either neighboring or distant) within the same hemisphere. They form bundles, which have been studied to greater or lesser degrees. The bundles that have received the most attention in tractography research are the cingulum, inferior fronto-occipital fasciculus, uncinate fasciculus, and superior and inferior longitudinal fasciculi. In our study we reconstructed the cingulum. The cingulum is a medial bundle located in the limbic system that crosses the cingulate gyrus. It is connected to the occipital, parietal and frontal lobes. Our DTI results show only the central part of the cingulum next to the corpus callosum while our fiber dissection results show a curved band of neural tissue located in the cerebral cortex, encircling the corpus callosum with parietal and occipital radiations (Figures 2, 3).

### Commissural fibers

Crossing the midline, commissural fibers connect homologous cortical areas. They are present in three bundles: the rostral and caudal commissures and corpus callosum. Only the latter was successfully reconstructed in our study. The corpus callosum is composed of fibers connecting neopallial areas. Forming the ceiling of the lateral ventricles, they cross the fibers of the corona radiata in the centrum semiovale. The corpus callosum can be divided into three parts: the central part (body) connects parietal and temporal areas; the rostral part (genu) connects frontal areas; and the caudal part (splenium) connects occipital lobes. The callosal sulcus separates the corpus callosum from the adjacent midline cortex, the cingulate gyrus (Figures 1A,C, number 8).

**Figure 4:**
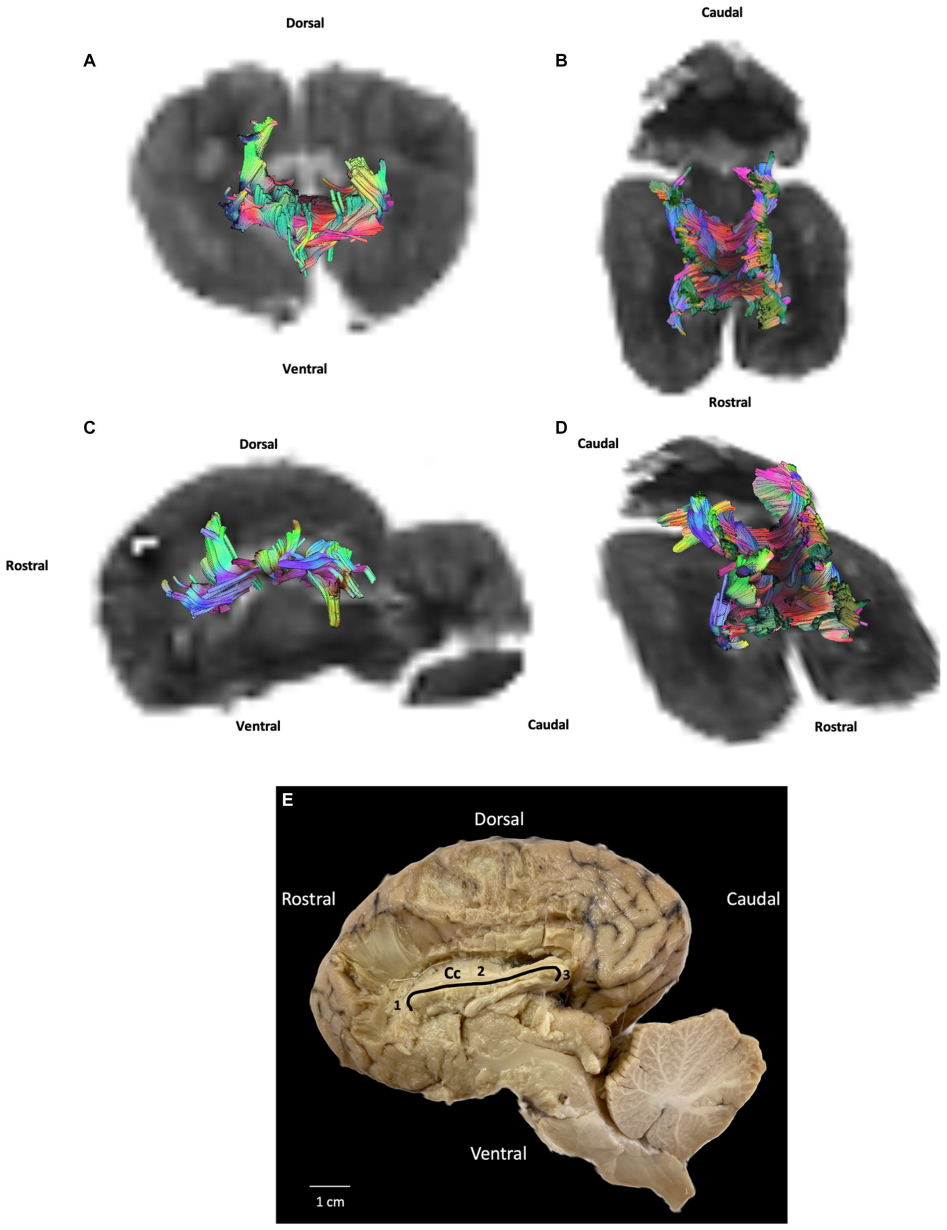
Example of commissural fibers (corpus callosum): **(A)** transversal plane – rostral view; **(B)** dorsal plane – dorsal view; **(C)** sagittal plane – left view; **(D)** oblique plane – dorsal view and **(E)** fiber dissection (underlined in black) using Klingler’s method. Cc: corpus callosum; 1: corpus callosum (genu); 2: corpus callosum (body); 3: corpus callosum (splenium). All tracts are overlaid on the b0 image obtained by DTI.

Whether in DTI or fiber dissection, our results show a large and extended corpus callosum throughout all the cortex (Figures 3, 4).

### Projection fibers

**Figure 5:**
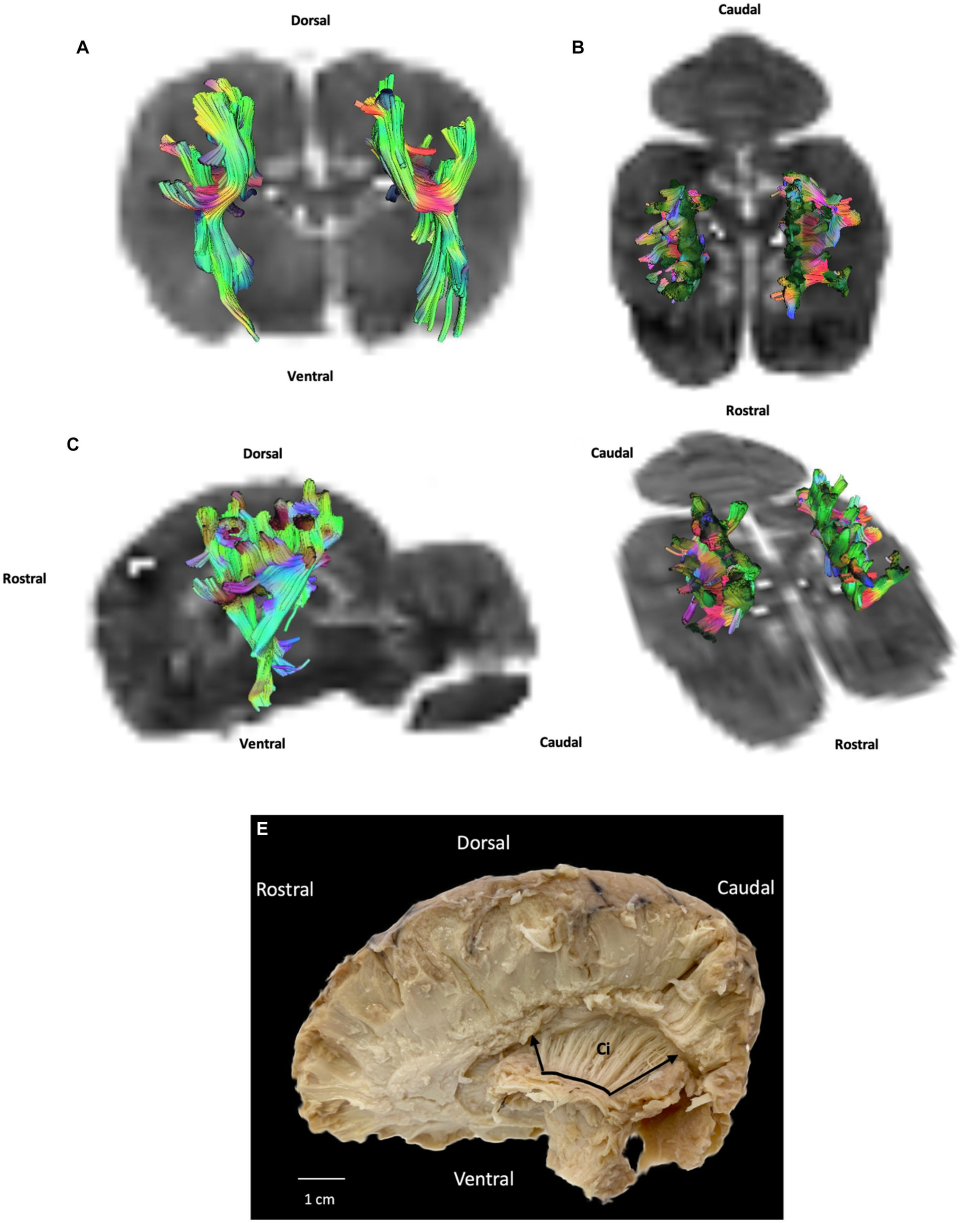
Example of projection fibers (internal capsule): **(A)** transversal plane – caudal view; **(B)** dorsal plane – dorsal view; **(C)** sagittal plane – left view; **(D)** oblique plane – dorsal view and **(E)** fiber dissection (underlined in black with margins framed by arrows) using Klingler’s method (cerebellum was removed). Ci: internal capsule. All tracts are overlaid on the b0 image obtained by DTI.

Projection fibers are responsible for connecting the cerebral cortex to other parts of the nervous system, such as the deep nuclei, brainstem, cerebellum, and spinal cord. The most significant complex of projection fibers is the internal capsule and corona radiata. This complex contains two types of fibers: corticopetal fibers connecting the thalamus and metathalamus to the cerebral cortex; and corticofugal fibers running from the cortex to various parts of the nervous system, not least the cerebellum (corticopontocerebellar tract), ventral rhombencephalon (corticobulbar tract), pons (corticopontine tract), mesencephalon (corticomesencephalic tract), and spinal cord (corticospinal tract). In our study we were unable to distinguish between these different tracts. Our results show the internal capsule situated in the inferomedial part of each cerebral hemisphere of the brain forming a V-shaped band of neural tissue located in the cerebral cortex, on either side of the corpus callosum with fibers from parietal and temporal cortex. The fiber dissection results also showed parietal and temporal radiations (Figures 3, 5).

## Discussion

First, it is important to note that no published data are available on DTI tractography of normal white-matter tracts in the one-humped camel. The present study yielded T2-weighted images of normal camel brain in 3 T with good resolution for the first time. It was essential to perform T2-weighted images, in order to understand the normal anatomy of white-matter fibers in the DT images. The anatomical description of the three fiber subgroups of association, commissural and projection fibers we established in the present study was closely correlated with descriptions found in dissection and anatomical textbooks. When we compared our results with those of other studies where DTI tractography and fiber dissection were performed to examine the brains of other species, we found both similarities and differences.

Concerning similarities, the corpus callosum in the one-humped camel consists of a flat bundle of fibers spanning part of the longitudinal fissure. This result is similar to that described in many large animals, especially in horses, large and small ruminants (20, 27, 40, 60). The internal capsule anatomy described in this study is similar to that of other large animals. The internal capsule carries sensory information from the thalamus to the cerebral cortex, allowing for the processing and interpretation of sensory inputs. It also carries motor information from the cerebral cortex to the spinal cord, enabling voluntary movements and coordinated motor responses. Given the unique adaptations of one-humped camels to desert environments, the internal capsule in these animals likely plays a role in supporting their specialized behaviors and physiological needs (61). For example, the internal capsule may contribute to the one-humped camel’s ability to navigate challenging terrains, since the internal capsule works as a relay station for the body’s motor fonction (61).

Differences concerning our DTI tractography findings relate to the cingulum. Our fiber dissection results show a curved band of neural tissue located in the cerebral cortex, encircling the corpus callosum with parietal and occipital radiations also described by Pascalau et al. in 2016. For instance, we failed to observe in DTI tractography theses parietal and occipital radiations. Moreover, only a small part of the cingulum attached to the corpus callosum could be reconstructed.

DTI tractography has a major advantage over *post mortem* anatomical techniques such as fiber dissection, as it allows for noninvasive dissection, yielding a three-dimensional representation of several white-matter tracts from a single imaging dataset (1, 3, 62). Due to its reliance on water molecule diffusion, DTI tractography provides an indirect assessment of axonal pathways. However, it struggles to accurately distinguish between crossing and branching patterns within a single voxel. As a result, tractography algorithms may produce erroneous connections or prematurely terminate tracked fibers, leading to anatomical inconsistencies between MRI-based reconstructions and dissection (53). As can be seen in our results, the current study is no exception. In our study, we acknowledge that due to the limitations mentioned earlier, such as difficulties in accurately characterizing crossing and branching patterns, certain white matter tracts were likely reconstructed incorrectly and exhibited anatomical inaccuracies. Specifically, we anticipate that the inferior fronto-occipital fasciculus, uncinate fasciculus, superior and inferior longitudinal fasciculi (associative fibers), as well as the rostral and caudal commissures (commissural fibers), and the corona radiata (projection fibers), may have been affected by these challenges and consequently misrepresented in our findings. The poor bad quality of the brain specimens can also explain the difficulty of some tracks reconstruction. This also explains the artefacts or halos present on T2-weighted images (Figure 1) and b0 images (Figures 2, 3). Moreover, even though manual ROI delineation can generate biases, we were unable to use automated segmentation, as there are currently no digital atlases of the one-humped camel brain. To minimize the biases of manual ROI delineation, we used T2-weighted images.

Given that the brain specimen used in this study was extracted from the skull and underwent formalin fixation, it is important to consider the potential impact of fixation-induced tissue changes and magnetic susceptibility artifacts at the tissue-air interface on the quality of MRI data. Postmortem DTI acquisitions require fixation to prevent autolysis, which can degrade the structural characteristics observed in *in vivo* conditions. Although the fixation process generally reduces overall water diffusion compared to *in vivo* conditions, its specific effect on diffusion anisotropy is not yet fully understood. Some studies indicate a decrease in anisotropy in fixed brains (63, 64), while others suggest that the fixative preserves relevant tissue microstructure (65, 66). To avoid these artifacts as far as possible, we immersed the brain in a bag filled with saline solution immediately prior to acquisition, as saline solution is isosignal with cerebrospinal fluid (51).

We chose to perform DTI reconstruction because it is the most popular and extensively used (and thus the most reproducible) fiber exploration technique (1). Future studies, however, should compared DTI with others algorithms on fiber tracking. For example, Q-ball imaging (QBI) reconstruction, in contrast to DTI reconstruction, takes multiple fiber orientations into account, rather than simply yielding an ellipsis. It could therefore prove particularly useful when multiple fibers cross within a single voxel (67).

We demonstrated that DTI tractography is possible in the *ex vivo* one-humped camel brain, allowing for the reconstruction of white-matter tracts. Scanning time (1 h) was compatible with anesthesia, and the T2-weighted and DTI parameters used in this study can be referenced for subsequent studies of the camel head. Finally, theses acquisition parameters could be easily adapted for *in vivo* studies, and it would be possible to reduce acquisition time, which is a limiting factor for the MRI examination of one-humped camels because anesthesia in large animals requires a lot of attention and is very often risky for the animal. Another limiting factor for *in vivo* studies is the requirement for high field MRI systems that can adequately support the weight of the animal being scanned. However, there are numerous MRI systems available that are capable of scanning the head of large animals *in vivo*, such as horses. Therefore, it appears feasible to use these existing MRI resources for scanning the head of a one-humped camel as well.

Furthermore, *ex vivo* imaging protocols necessitate the use of larger b-values (3,000 in our study). This is done to counterbalance the significant reduction in the apparent diffusion coefficient observed *ex vivo* and thereby maintain diffusion contrast. Additionally, the T2 relaxation time is substantially reduced in *ex vivo* conditions, which limits the available window for data acquisition. Consequently, stronger gradient magnitudes are required to minimize echo times and preserve the signal-to-noise ratio in *ex vivo* imaging (68, 69). In the present formaldehyde-fixed *post mortem* study, we had to run five series of acquisitions (5 × 12 min) on average in order to achieve a sufficient signal, but this would not be necessary for *in vivo* studies, given that the signal would naturally be stronger. Fewer iterations of each direction would be acquired, thereby possibly reducing acquisition time. However, only one sequence was tested in our study, so it would be necessary to compare this one with any other potential sequences.

## Conclusion

The results of the present study show the reconstruction of the most representative tracts in the one-humped camel brain using DTI data acquired *post mortem*. Cingulum, corpus callosum and internal capsule could be reconstructed. Further DTI studies are needed to improve and validate these findings and to produce a tractography atlas for future neuroimaging research in the one-humped camel.

## Data availability statement

The raw data supporting the conclusions of this article will be made available by the authors, without undue reservation.

## Ethics statement

The animal studies were approved by the Comité d’Ethique Science et Santé Animales – Toulouse – France. The studies were conducted in accordance with the local legislation and institutional requirements was obtained from the owners for the participation of their animals in this study.

## Author contributions

BC and GM drafted the manuscript. NP, AA, RP, AM, KE, SK, BB, and CM helped to establish the experimental procedures. GA and GM jointly supervised this work. All authors contributed to the article and approved the submitted version.

## Conflict of interest

The authors declare that the research was conducted in the absence of any commercial or financial relationships that could be construed as a potential conflict of interest.

## Publisher’s note

All claims expressed in this article are solely those of the authors and do not necessarily represent those of their affiliated organizations, or those of the publisher, the editors and the reviewers. Any product that may be evaluated in this article, or claim that may be made by its manufacturer, is not guaranteed or endorsed by the publisher.
