# Supplementary material for: Diffusion tensor imaging tractography in the one-humped camel (Camelus dromedarius) brain
Source: Front Vet Sci. 2023 Aug 15;10:1231421. doi: 10.3389/fvets.2023.1231421 (PMC10464492; doi:10.3389/fvets.2023.1231421)
Supplement: Supplementary file 1 [file Data_Sheet_1.PDF]

Median plane

FL: Frontal Lobe ; PL: Parietal lobe ; OL: Occipital lobe ; IA: interthalamic adhesion; III: third ventricule ; IV: fourth ventricule; Lat V: lateral ventricle

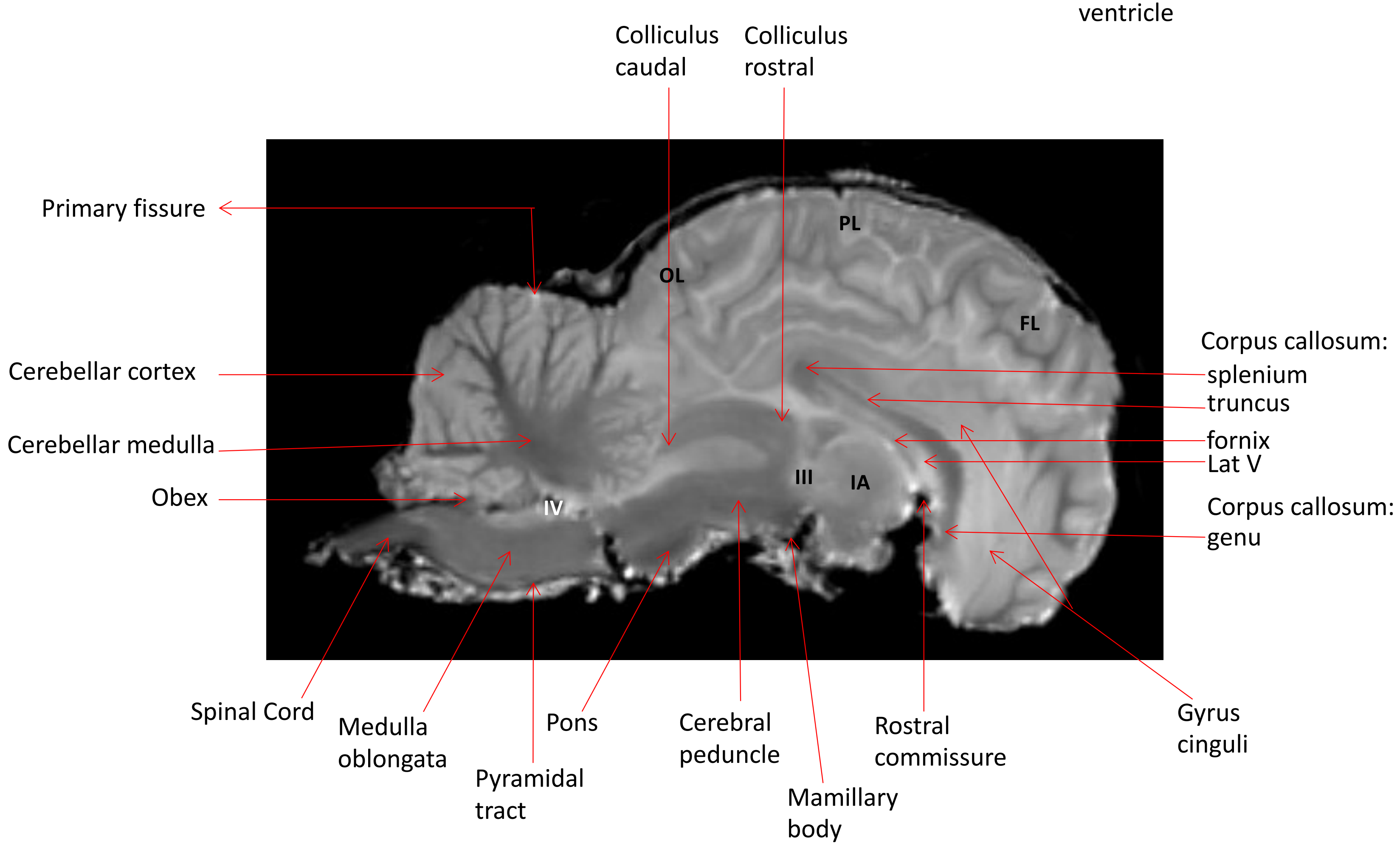

Dorsale planes

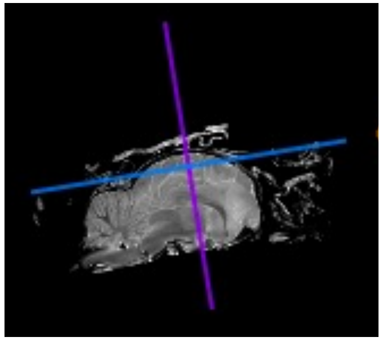

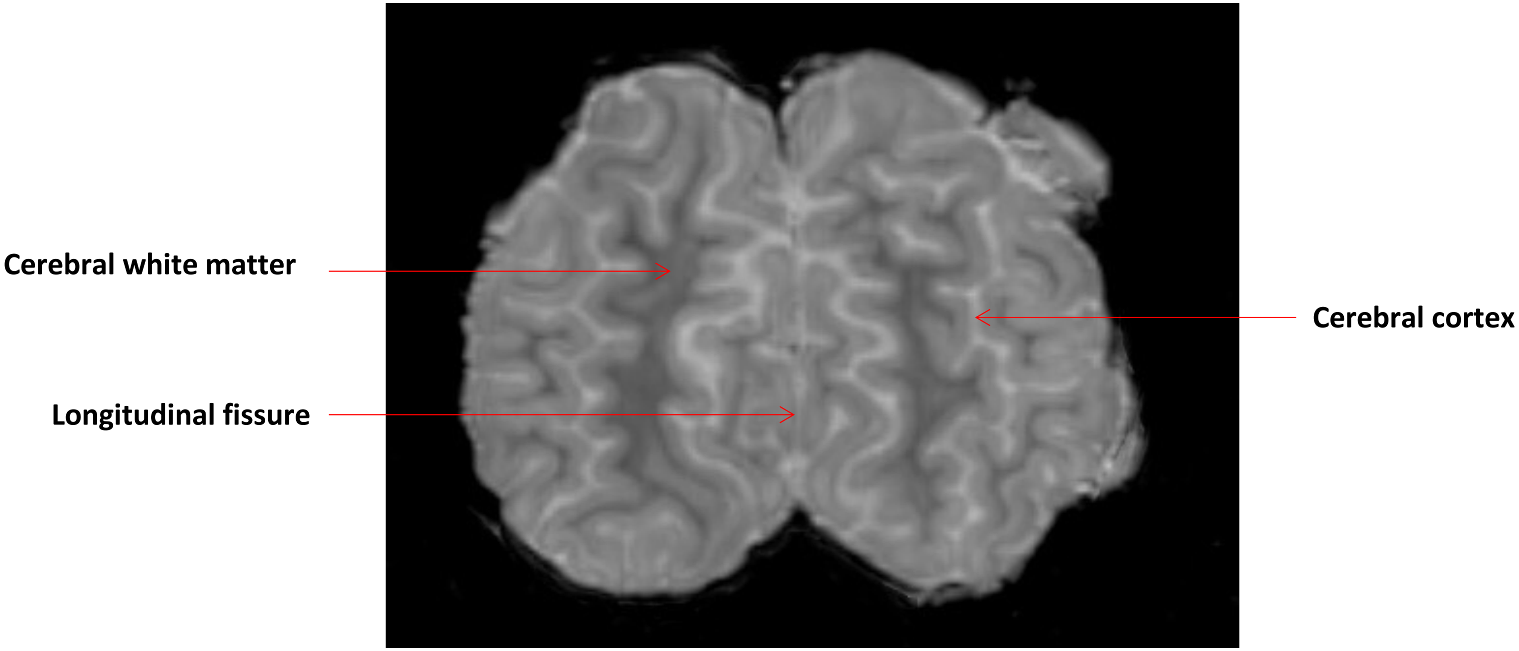
Cerebral white matter
Longitudinal fissure
Cerebral cortex

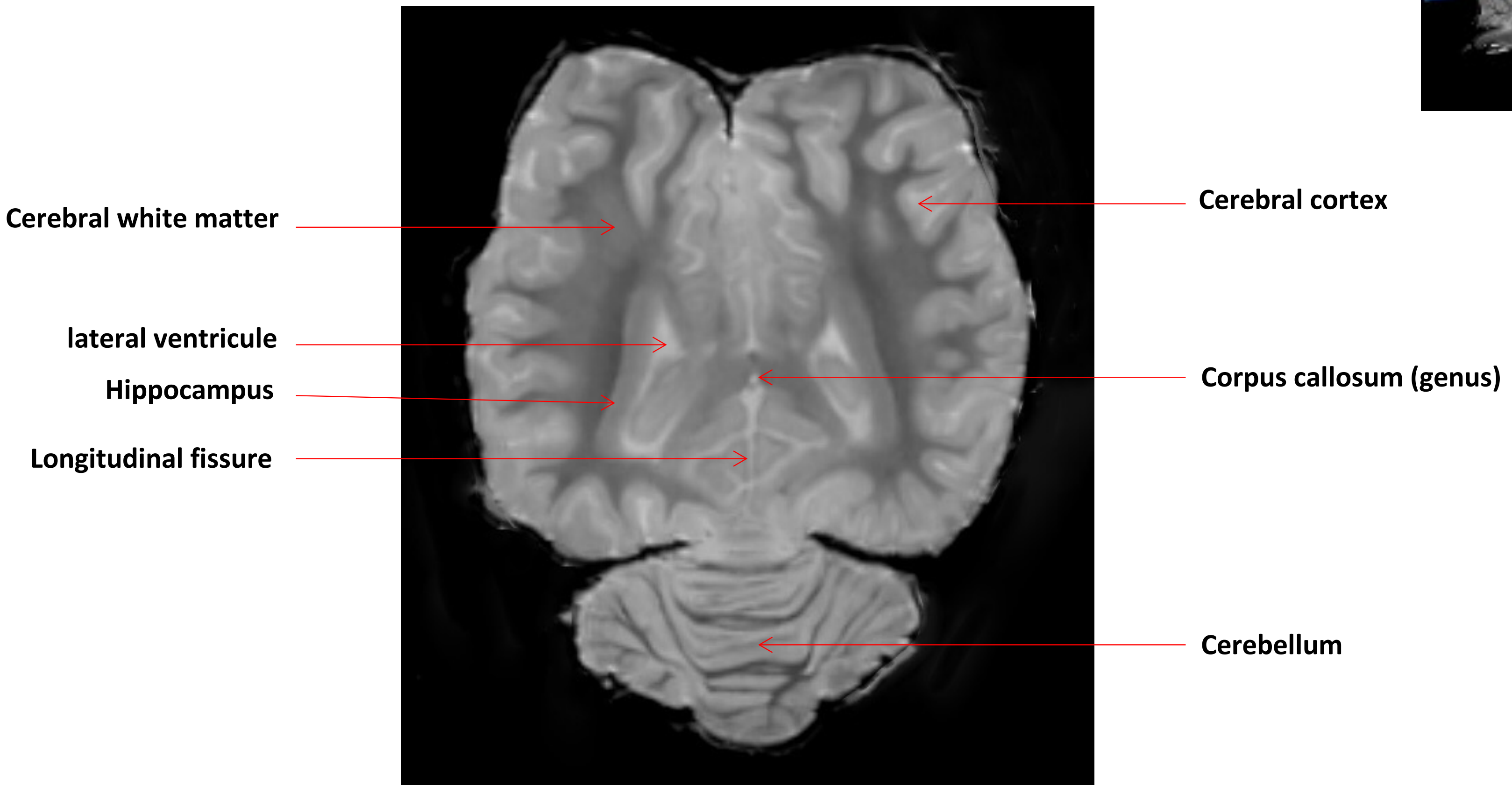

Cerebral white matter
lateral ventricule
Hippocampus
Longitudinal fissure
Cerebral cortex
Corpus callosum (genus)
Cerebellum

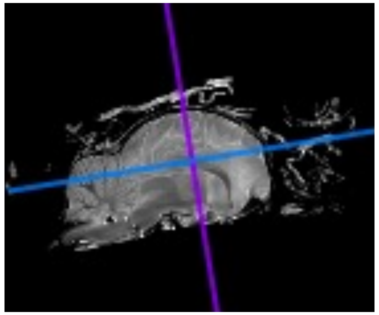

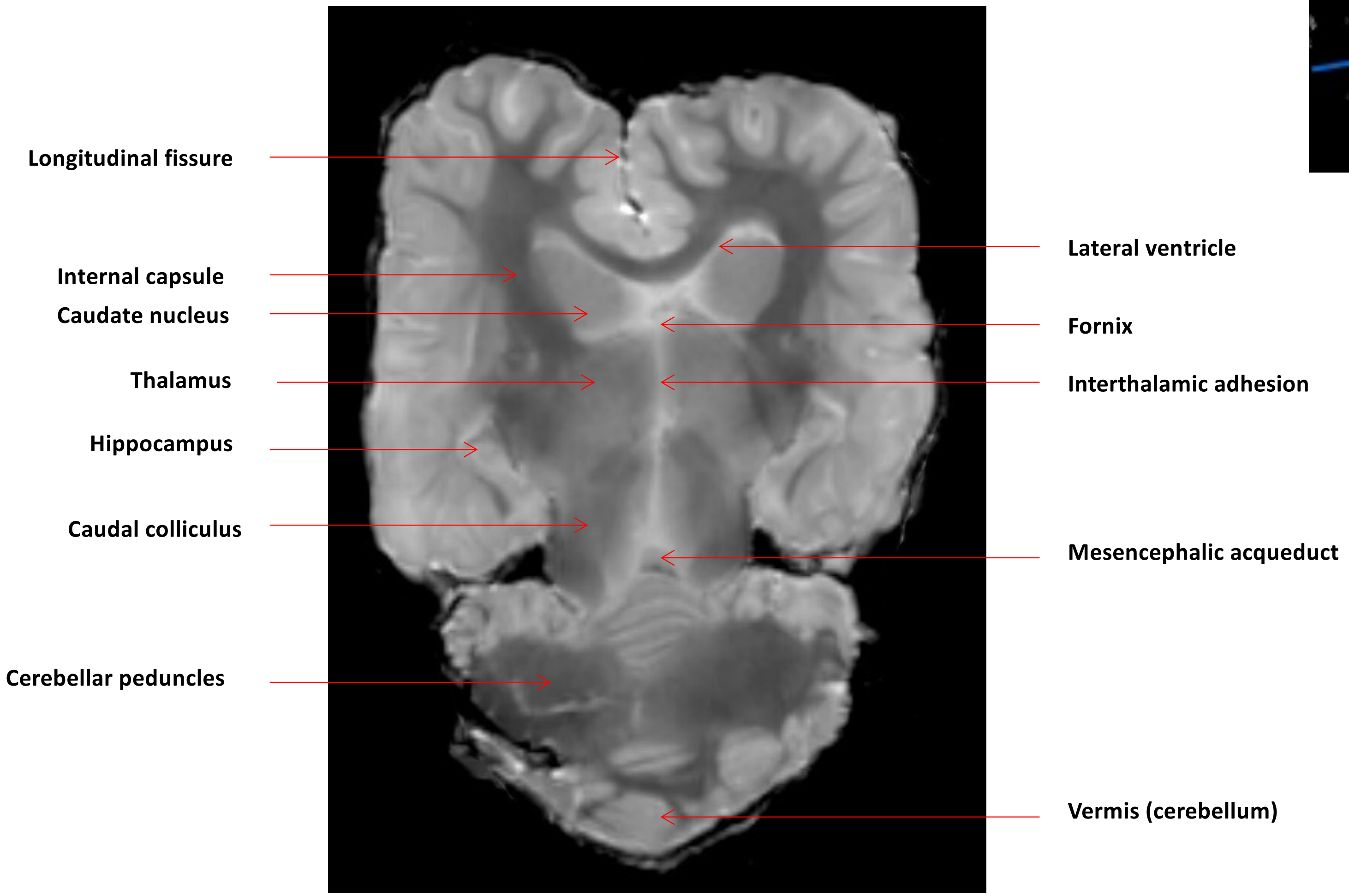
Longitudinal fissure
Internal capsule
Caudate nucleus
Thalamus
Hippocampus
Caudal colliculus
Cerebellar peduncles
Lateral ventricle
Fornix
Interthalamic adhesion
Mesencephalic acqueduct
Vermis (cerebellum)

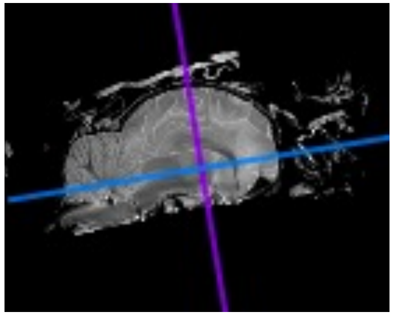

Transversal planes (from caudal to rostral)

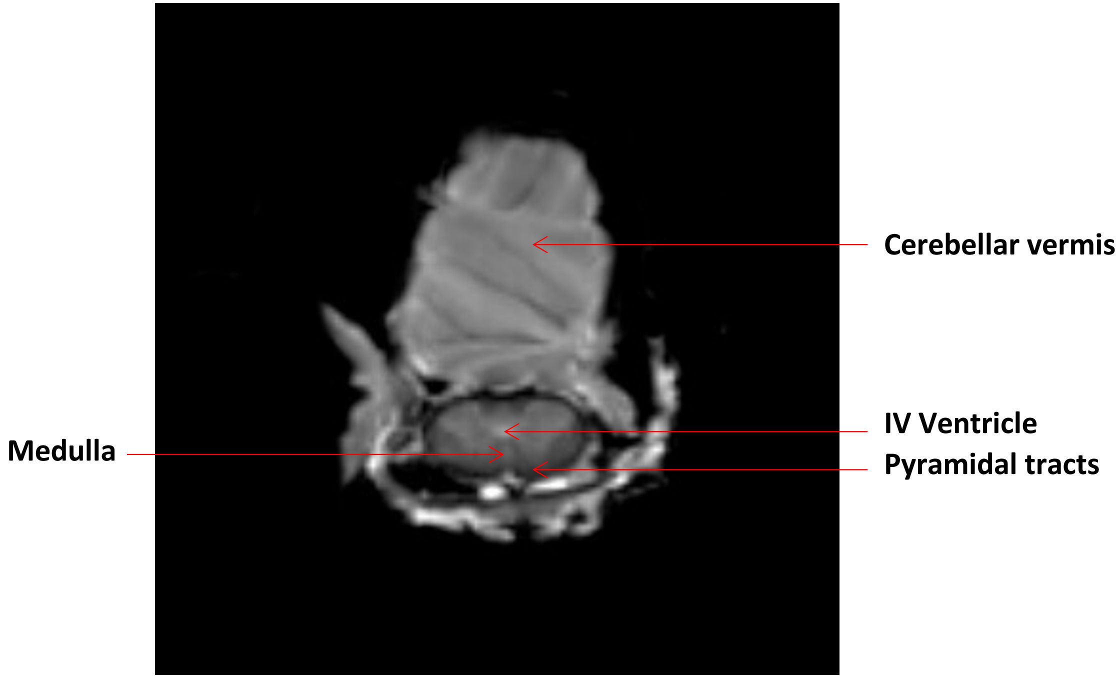
Cerebellar vermis
Medulla
IV Ventricle
Pyramidal tracts

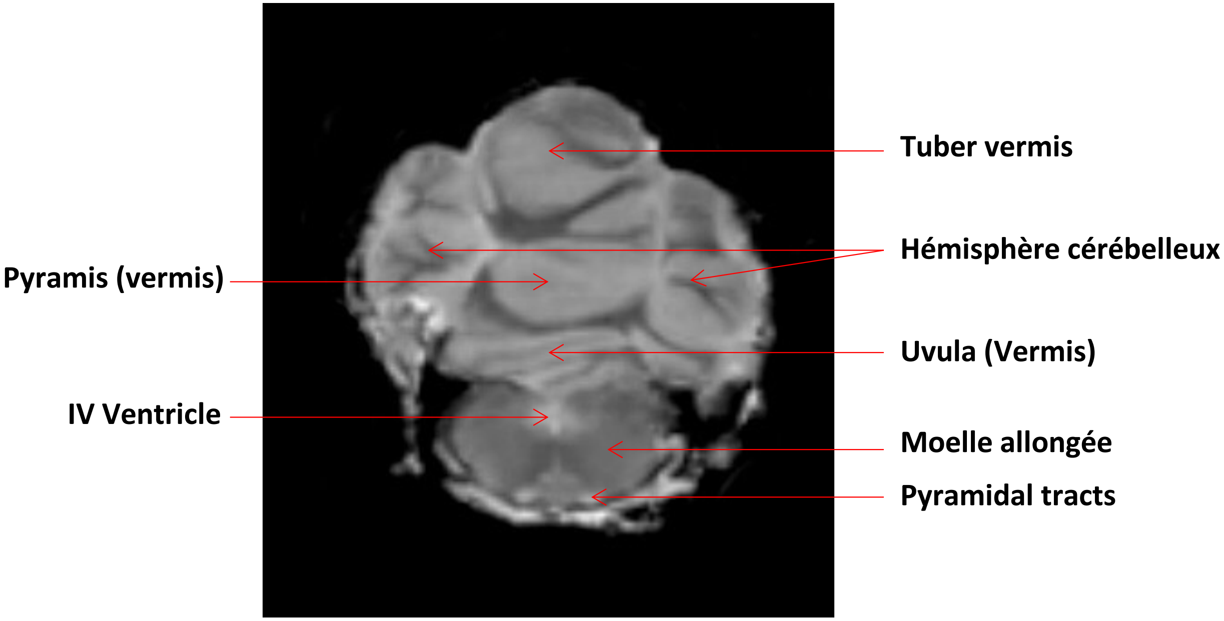
Tuber vermis
Hémisphère cérébelleux
Pyramis (vermis)
Uvula (Vermis)
IV Ventricle
Moelle allongée
Pyramidal tracts

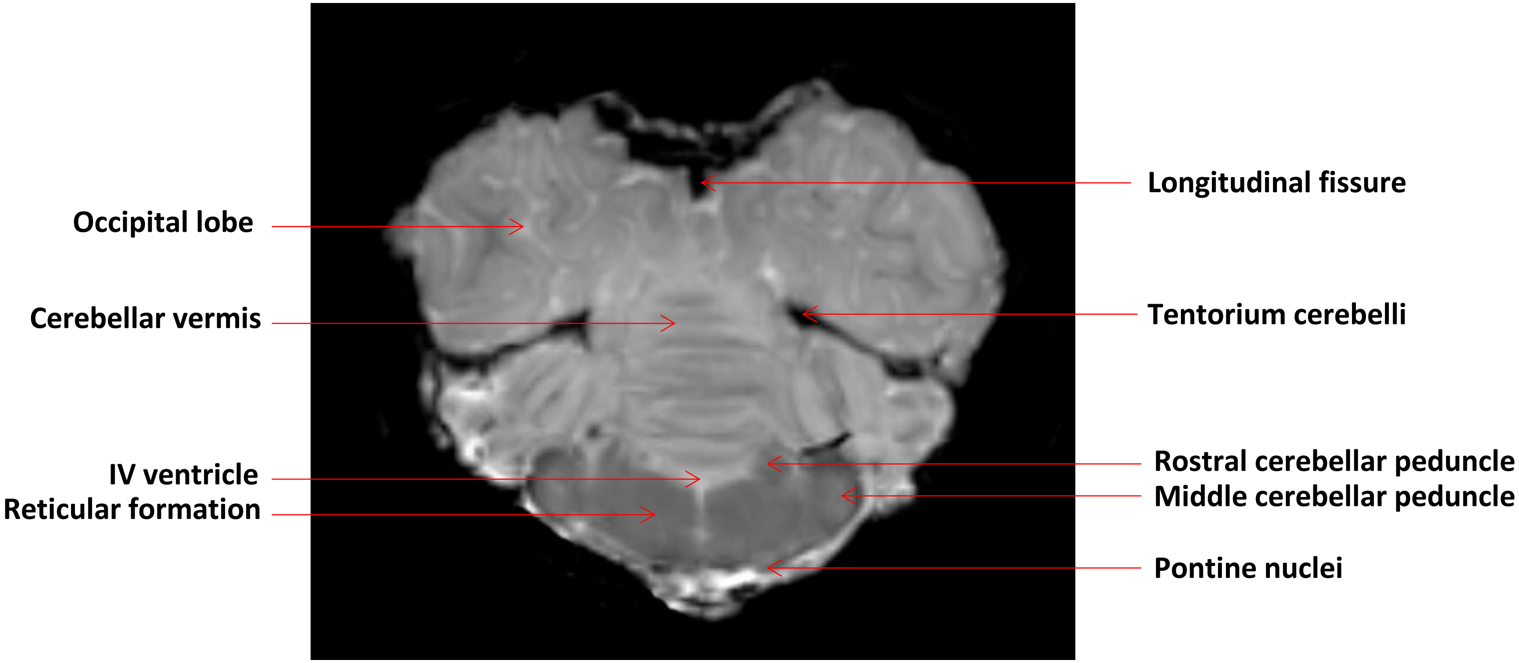

Occipital lobe
Cerebellar vermis
IV ventricle
Reticular formation
Longitudinal fissure
Tentorium cerebelli
Rostral cerebellar peduncle
Middle cerebellar peduncle
Pontine nuclei

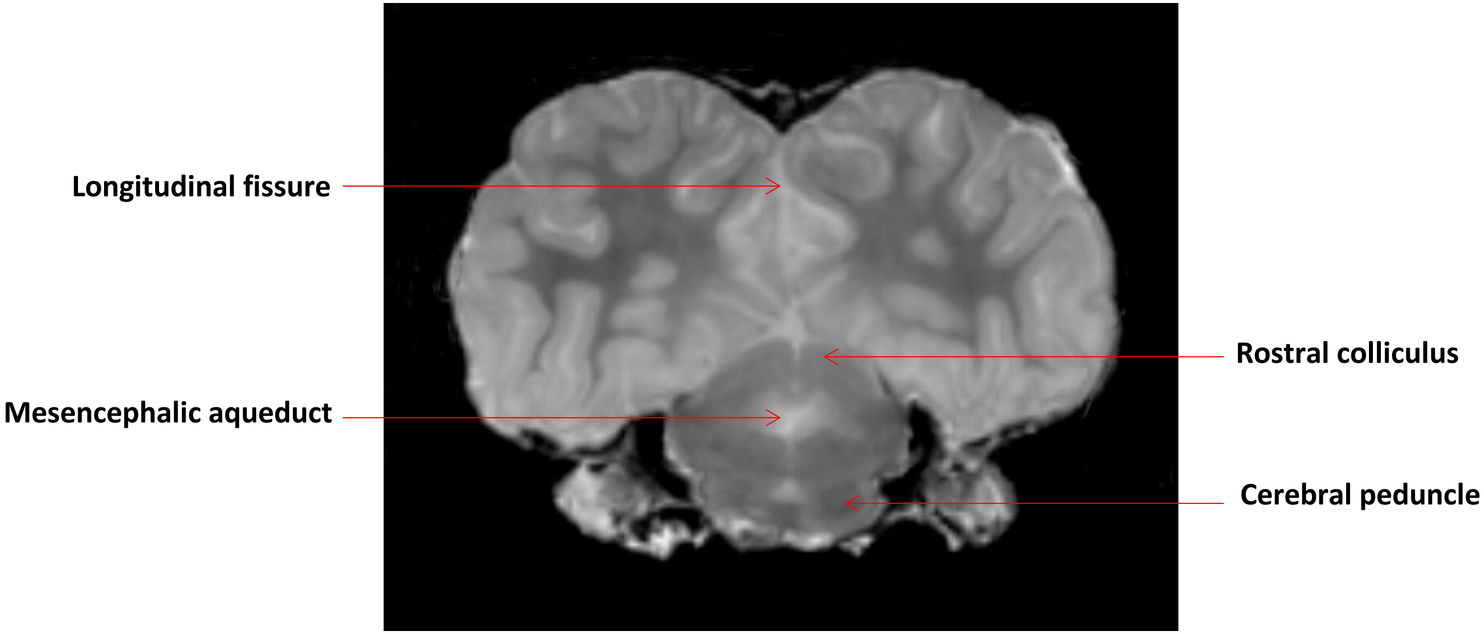
Longitudinal fissure
Rostral colliculus
Mesencephalic aqueduct
Cerebral peduncle

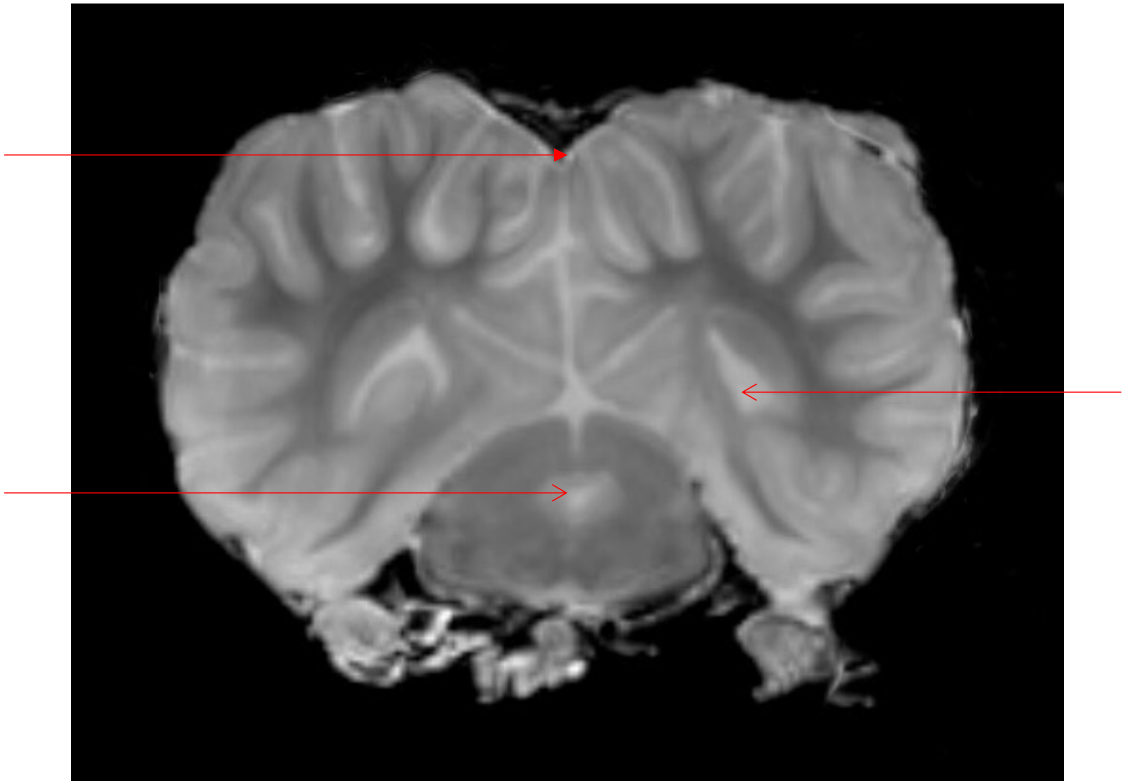
Longitudinal fissure
Lateral ventricle
Mesencephalic aqueduct

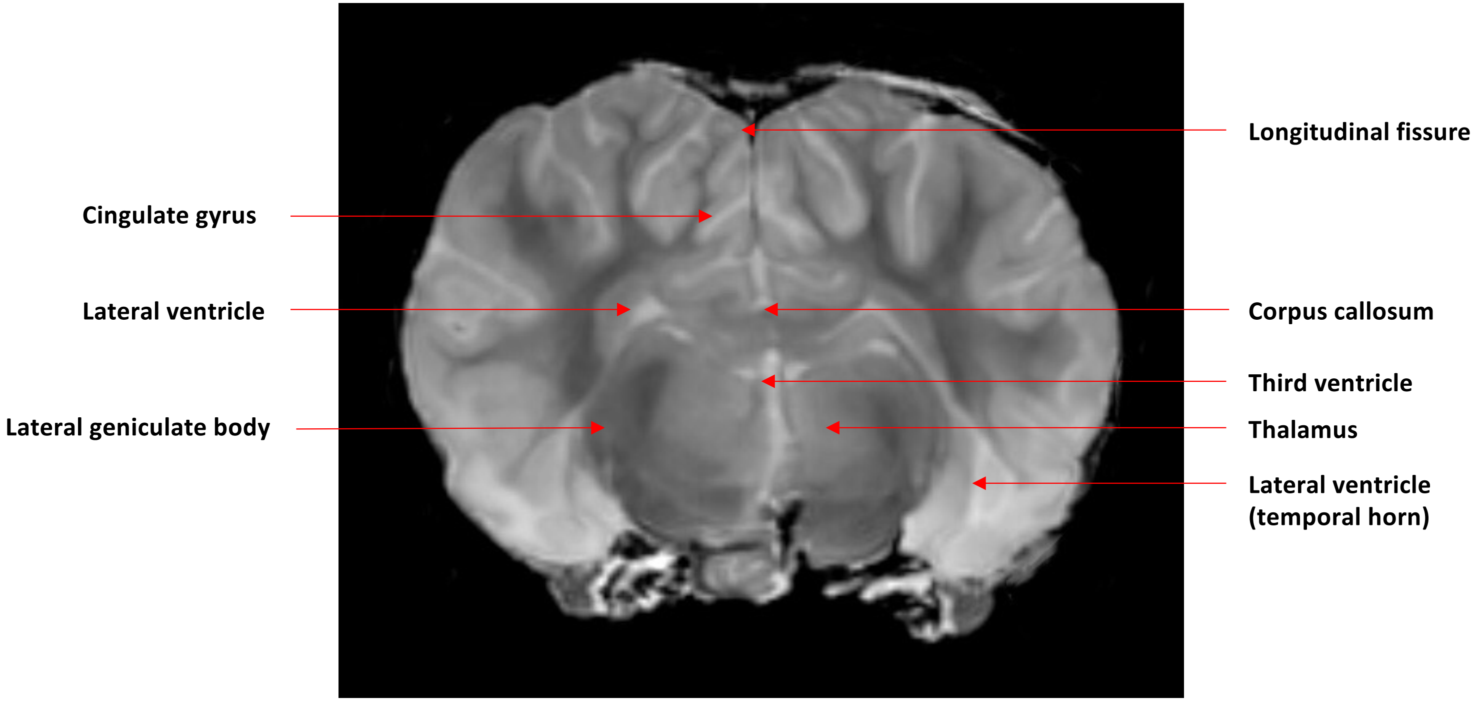

Longitudinal fissure
Cingulate gyrus
Lateral ventricle
Corpus callosum
Third ventricle
Lateral geniculate body
Thalamus
Lateral ventricle
(temporal horn)

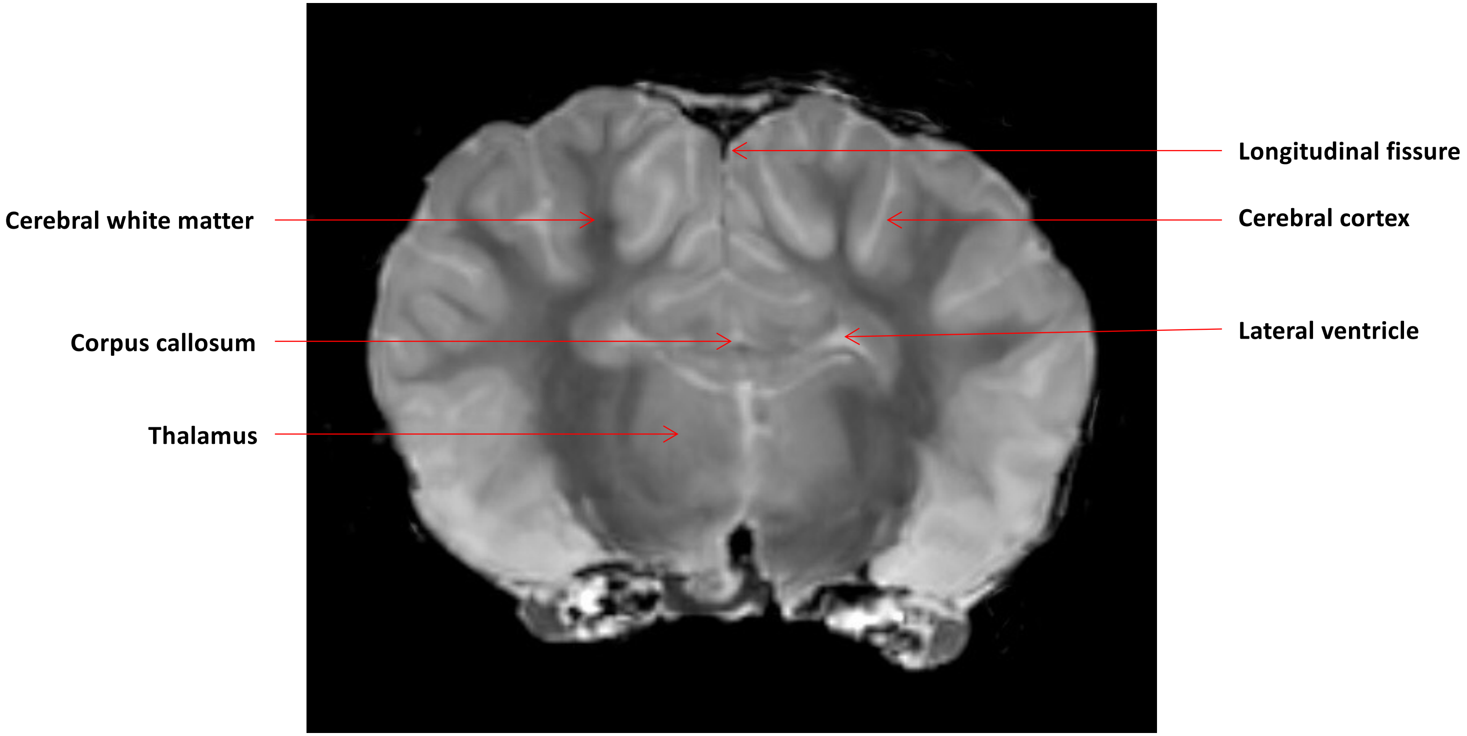
Longitudinal fissure
Cerebral white matter
Cerebral cortex
Corpus callosum
Lateral ventricle
Thalamus

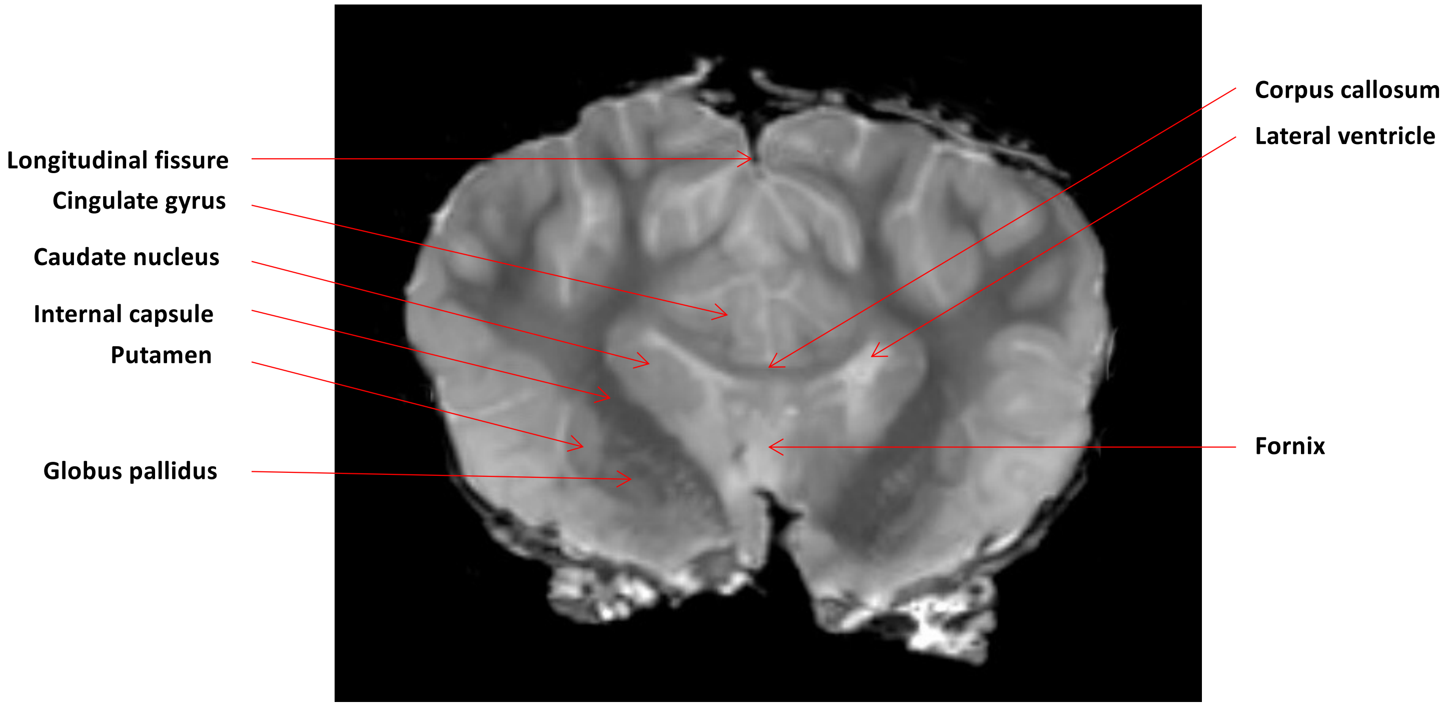
Longitudinal fissure
Cingulate gyrus
Caudate nucleus
Internal capsule
Putamen
Globus pallidus
Corpus callosum
Lateral ventricle
Fornix

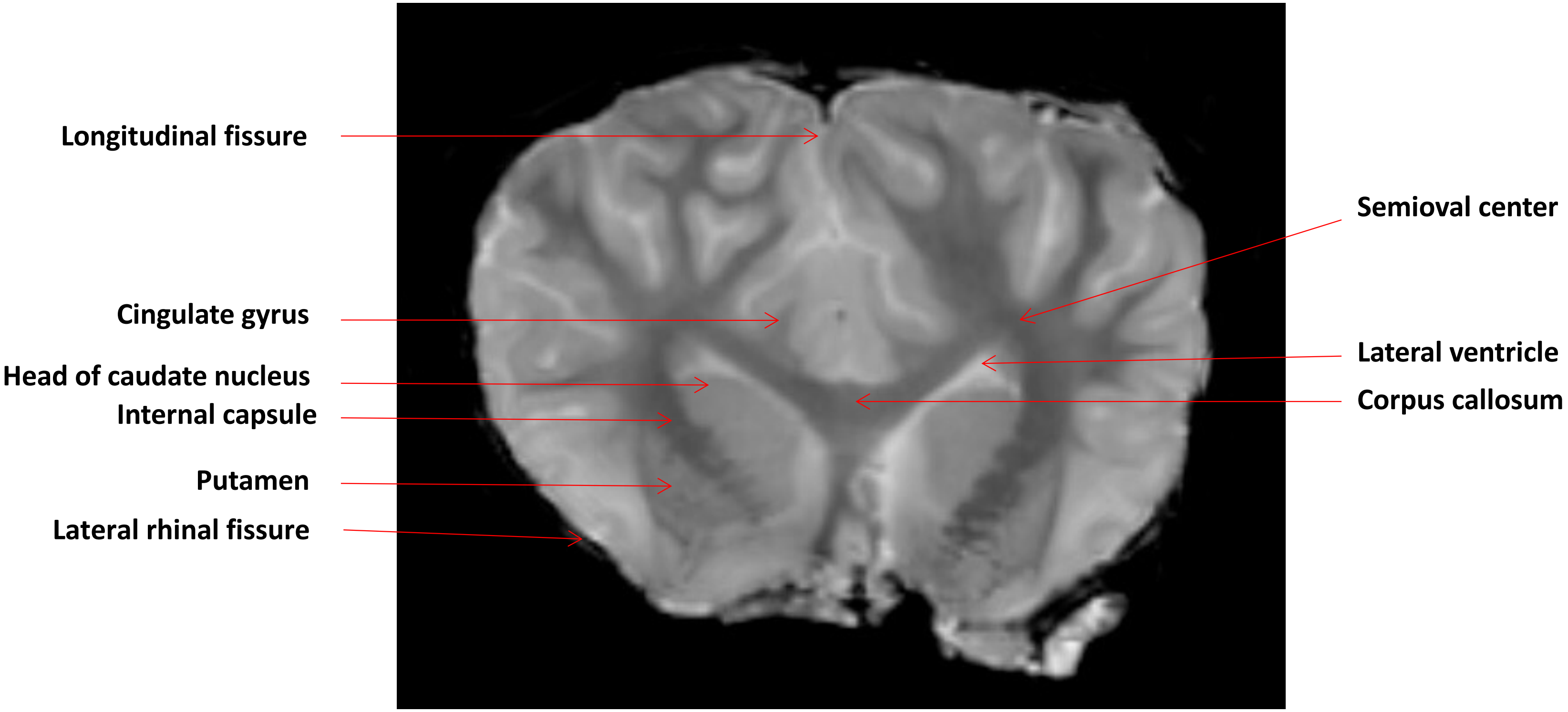
Longitudinal fissure
Cingulate gyrus
Head of caudate nucleus
Internal capsule
Putamen
Lateral rhinal fissure
Semioval center
Lateral ventricle
Corpus callosum

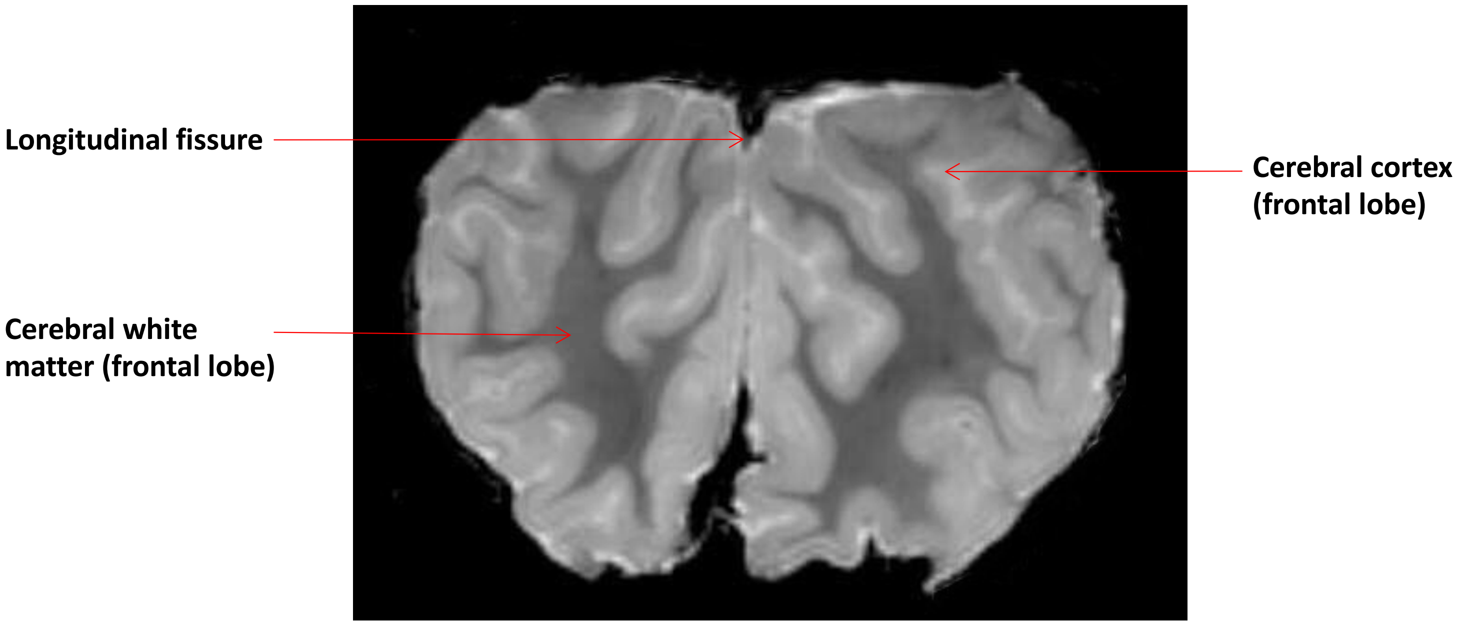
Longitudinal fissure
Cerebral cortex
(frontal lobe)
Cerebral white
matter (frontal lobe)
